# Monthly Report of Diseases

**Published:** 1800-11

**Authors:** 


					Monthly Report of Diseases. 3^3
MONTHLY REPORT of DISEASES,
Admitted under the Care of the Physicians ot the I'insbury
Dispensary, St. John's Square, Clerkenwell.
From September 20 to Oftober 20, 1800.
Continued Fever - - - 60
Quotidian ----- 1
Pneumonia ----- 7
Small-Pox" ----- 1
Rheumatifm - - - - - 15
Cholera ------ 6
Diarrhoea - -- -- -13
Pyfentery ----- 29
Menorrhagia - - - - 5
Amenorrhea and Chioroiis 10
Scirrhus Uteri - - - - 2
Hasmorrhois - 1
' Erylipelas - - - - I
Optnalmia ----- 1
Cephatea ----- 3
Dropfy - ^
Dyfpepfia, Gafcrodynia, and
En:erodynia - - - - li
Aithcnia ----- 16
Catarrh - - - - - 5
Pleurodyne ----- 6
Cough and Dyfpnoea - - 11
Jaundice ----- 2
Nephralgia ----- 1
Phthi is Pulironalis 6
Hypochondriafis - - -2
Scrophula ----- 2
Prolapfus Ani - - 1
He&ica Senilis - - - - 1
Chronic Eruptions - 11
Acute difeaies of Infants - 12
The
\
394- Observations on Disease! in London*
The cholera has now nearly difappeared, and the diarrhoea and
dyfentery have been lefs frequent than in the preceding month j but
the latter difeafe is become more fevere, and has aflumed a more
decided chata&er. The fymptomatic fever, however, by which '
it is generally accompanied, has been very flight, and in fome in-
ftances fcarcely perceptible. In thofe perfons whofe vifcera have
been injured by a courfe of hard drinking, or by previous attacks
of fimilar complaints in a hot climate, the dyfentery proves Angu-
larly obftinats, and, if it does not terminate in death, leaves them
in an extreme ftate of debility and emaciation, with a moft exqui-
fite tendernefs siid irritability of the alimentary canal/ and it is
only by a ftrift attention to diet and regimen, that they gradually
recover a tolerable {hare of health and ftrength, Which require ever
afterwards the greatefl care for their prefervation. . Notwithftand-
ing the prohibition of opium in this difeafe, by feveral refpeftable
" authors, its daily adminiftration, after the operation of a purga-
tive, fo far from producing any bad confequences^ feemed to pro-
mote the cure, and invariably afforded great eafe and comfort to
the patient. It is better that it (hould be combined with ipecacu-
anha, but this perhaps is of little importance.
The cafes of pneumonia were none of them formidable^ Ge-
neral blood-letting was not once required. The application of
leeches to the thorax, and the repeated ufe of blifters, with anti-
monials, calomel, and opiates, proved fully adequate to the .cure.
The rheumatifm was moftly of the chronic kind. In feveral af-
flicted with it, a pill containing one grain of opium, and one of
calomel, taken daily for about ten days, was of the molt eminent
fervice, after fudorifics, bark, and guaiacum had been adminiftered
for a long time without any benefit whatever.
It is feldom that an intermittent fever appears in our lift. This
difeafe, which in the time of Sydenham was one of the principal
epidemics of London, is now rarely to be met with. When it oc-
curs, it is commonly in labourers who have lately returned from the
fenny counties, where they had been engaged in the bufinefs of the
harvell.
The cafe of ptyalifm, infertcd in the catalogue of the laft
month, being attended with circumftances rather unufual, is per-
haps worthy of notice. A man about fifty years of age, of a
ftrong and plethoric conftitution, has.for thefe fix years part been
affe&cd with a preternatural difcharge of faliva, to the amount
of a pint in the twenty-four hours, and of a very vifcid conflu-
ence. It takes place chiefly in the morning after rifmg from bed,
when he finds a fenfe of fullnefs and uneafinefs about his head,
which is immediately relieved by the fpitting. His health is toler-
ably good, and the other fecretions are natural. There appears to
have been no obvious caufe of his complaint, but he mentions that
he had a very fevere fever about fix months before he perceived it.-
He is in the.habit of fmoaking tobacco, l>ut he has found that the
omiffion of the praftice for a confiderable time, occafions no alter-
ation in the difcharge. What" is remarkable, he is enabled, by
a voluntary effort perfifted in for a few d^ys? to. reduce the fecre-
Observations on Diseases in London.
395
tion to its natural quantity. At the fame time, however, he be-
comes affefted with head-ach and giddinefs; his appetite fails
him; and the ftc.mach and abdomen are painful and much dif-
tended. Thefe fymptoms are then relieved by a profufedifcharge
of blood from the hemorrhoidal veins. Bv again encouraging
the fecretion of faliva, the haemorrhoids ceafe, and he regains
his ufual health. He has confulted a variety of phyficians and
medical praftitioners, and has undergone repeated courfes of me-
dicine for the alleviation of his complaint.?Purgatives, fudori-
fics, tonics, diuretics, mercurials, ilfues, and a vegetable diet have
all been tried in vain. Blood letting to the amount of ten ounces
puts a ftop to the difcharge for fix weeks or two months, without
his experiencing the above mentioned morbid fymptoms. But as
the repeated lofs of blood muft neceffarily weaken him much more
than the increafed fecretion of faliva, it is furely a remedy more
to be dreaded than the difeafe.
Although the continued fevers in the laft month have exceeded
thofe in the preceding by a fmall number only, we are concerned
to ftate that the proportion of mortal cafes has very confiderably
increafed. There has not appeared, however, any additional vio-
lence or malignity in the fymptoms; in almoft all the inftances of
fatal termination, the patients died at a very extended period of
the difeafe, their ftrength being rather gradually exhaufted by its
duration than overpowered by its force. One young perfon expired
at the end of the lixth week in fuch an extreme ftate of emacia-
tion, that, on an infpe&ion of the corpfe, one would have fup-
pofed her to have been theVidtim of the moll lingering confump-
tion. About the end of the third week, a complete jaundice took
?lace ; but it had almoft entirely difappeared before her death,
'he febrile heat fubftded at the beginning of the fourth week;
the pulfe alfo became lefs quick, and did not regain its former
rapidity till a day or two before {he died. It happens perhaps
more commonly than is imagined, that in thefe fevers the heat of
the fkin is not raifed above the natural ftandard In one inftance
of this kind, which occurred to the writer of this article, the
pulfe beat only feventy-two ftrokes in a minute : thus the two cir-
cumftances which have been generally regarded as the moil: efien-
tial charatterittic of the prefence of fever were entirely wanting.
The contagion from which thefe fevers originate is conftantly
generated and preferved in the dirty crowded dwellings of the
poor. Several circumftances, but principally certain ftates of the
air, favour its formation and extenfion, and influence its aftivity
and virulence. Daring the laft twelve months, indeed, there has
unfortunately exifted a caufe much more powerful in promoting
the operation of contagion on the lower clai's, than any properties
of the atmofphere?a lamentable deficiency of the common arti-
i cles of nourishment. To this, which of itfelf is well known to
be a principal pre-difpofing caufe of the typhus fever, may be
added the gloomy and deprelfed ftate of mind, which parents at
leaft muft experience, when furrounded by a hungry offspring,
they find themfelves unable to fatisfy their urgent demands for
bread. Hence alfo the recovery of convalelcents is unufually
flow; they are more liable to relapfes, and often fink into many
396
Observations on Diseases in London.
lingering difordete, of which debility is the principal caufe and
fymptom.
The loathfome circumftances attending the crowded Jhabitations
of the poor in large cities, and their abfurd and deftru&ive me-
thods of domeftic economy, which prove a never failing fource of
febrile contagion ; their extreme mifery and fufFering when once'
a fever has taken pofleflion of their families, and the dreadful ra^
vages it occafions among them, have lately been very accurately
and pathetically defcribed by three phyficians, whole profellional
avocations have, for a number of years, led them to be extenfivejy
acquainted with the condition and difeafes of the poor in the me-
tropolis, and in the populous towns of Manchefter and Liverpool,
and whofe humanity and benevolence render, them not lefs an ho-
nour to their race than their learning and medical fkill to their pro-
feifion.*
In confequence of the forcible reprefentations of Dr. Ferriar of
Iflar.chefter, feyeral refpeftable gentleman of tha^t town formed
themfelves into a board of health, and opened a fubfeription for
the fitting up an hofpital or houfe of recovery, for the reception of
the poor, ill of contagious fever. Into this houfe the patients are
removed as early as poffible. Their inftefted garments are taken
off, to be purified and reftored to them on their difmiflal; their per-
fons are made clean by lukewarm water ; they are put into verrtila-
ted wards, where they have the advantage of medical attendance,
medicines, and nurfing. In the mean time meafures are taken to
defiroy the contagion in the habitations^they have left. By white-
wafhing, ventilation, foap and water, flaked lime, and the vapour
of the nitric acid, their tainted apartments are completely purified
and made fit to receive their now wholefome tenants with fafety.
?The advantages which have refulted from this admirable in-
ftitution have exceeded the molt fanguine hopes of its benevolent
founders. In feveral of the ftreets of Manchelter, where the fever
was wont to rage in its moil deftru&ive form, it is now nearly an-
nihilated.
Wc are happy to hear that fome public-fpirited individuals have
it in contemplation to attempt the eftablilhment of fimilar inftituti-
cytis in this metropolis. Every medical man, who is in the habit of
vifiting the fick poor, mull give his teftimony for the neceffity of
fuch a plan; and when the inhabitants in general ftiall be made
fully acquainted with the alarming magnitude of the evil, and the
eafy and erreciual means of greatly diminifliing, if not of abfo?
Intel v remov ing it, we have good reafon to hope that the noble fpi-
rit of charity, by which they are diftinguiflied throughout Europe,
will not long delay to accomplilh an object more important, and
fraught with more ufeful confequences to the community, than any
which for a long time pail has engaged the attention of the bene-
volent. W. W. '
? ? ? JR- I
_ . _ -
* See Dr. Ferriar's Med. Hiftories and Refle&ions, Vols. 1, 2, and 3 ; J
Dr. Cume's Med. Reports on Fever, See. and Dr. Willan's Account
uf Difeafes in London in our Journal tor April, iSoo.

				

